# P-753. Characterizing Multidrug-Resistant *Acinetobacter baumannii* Infections among Wartime Casualties

**DOI:** 10.1093/ofid/ofae631.949

**Published:** 2025-01-29

**Authors:** Stone A Frankford, Laveta Stewart, Erica Sercy, Matthew Igo, Andrew Wyatt, M Leigh Carson, Wesley Campbell, Katrin Mende, David Tribble, John L Kiley

**Affiliations:** Brooke Army Medical Center, San Antonio, Texas; Infectious Disease Clinical Research Program, Henry Jackson Foundation, Bethesda, Maryland; Infectious Disease Clinical Research Program, Department of Preventive Medicine and Biostatistics, Uniformed Services University of the Health Sciences; Henry M. Jackson Foundation for the Advancement of Military Medicine, Inc., Washington, District of Columbia; Infectious Disease Clinical Research Program, Bethesda, Maryland; Landstuhl Regional Medical Center, Landstuhl, Rheinland-Pfalz, Germany; Infectious Disease Clinical Research Program, Department of Preventive Medicine and Biostatistics, Uniformed Services University of the Health Sciences, Bethesda, MD, USA, Bethesda, MD; Walter Reed National Military Medical Center, Bethesda, Maryland; Infectious Disease Clincial Research Program, JBSA Ft Sam Houston, Texas; Infectious Disease Clinical Research Program, Department of Preventive Medicine and Biostatistics, Uniformed Services University of the Health Sciences, Bethesda, MD, USA, Bethesda, MD; BAMC, San Antonio, Texas

## Abstract

**Background:**

*Acinetobacter baumannii* (ACB) emerged as a key pathogen during the war in Iraq and was designated as a serious threat in the 2013 Centers for Disease Control’s antimicrobial resistance report. We describe the characteristics of wounded military personnel with ACB infections.

**Figure:**
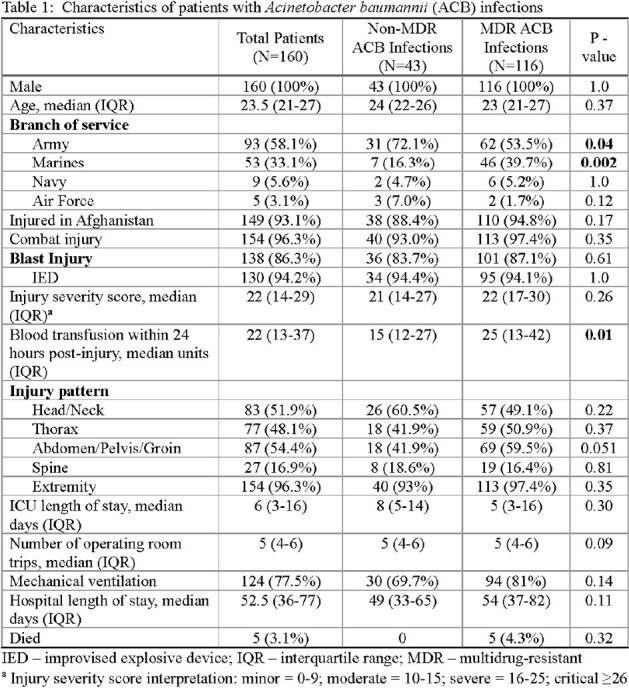

**Methods:**

Data were obtained from the Trauma Infectious Disease Outcomes Study, an observational study of US service members injured in Iraq and Afghanistan (6/09-12/14). Patients who had ≥ 1 positive infecting ACB isolate were assessed. Multidrug resistance (MDR) was defined as resistance to ≥ 3 classes of antimicrobials or production of extended-spectrum β-lactamase or carbapenemase. Categorical variables were compared by Chi-square or Fisher’s Exact and continuous variables by Mann-Whitney U.

**Figure:**
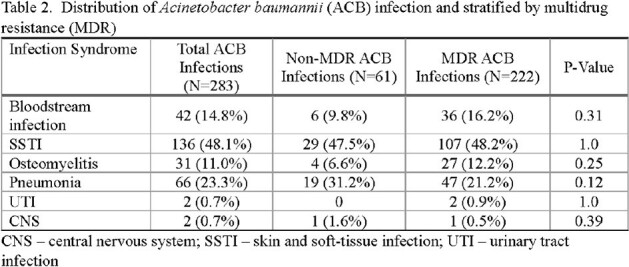

**Results:**

Among 160 patients with ACB infections, 116 (73%) and 43 (27%) had MDR and non-MDR ACB, respectively. All were male with a median age of 23.5 years and the majority sustained injuries in combat (96%) in Afghanistan (93%) (Table 1). Blast was the primary injury mechanism (86%), resulting in high injury severity. There was a total of 283 ACB infections with 222 (78%) MDR and 61 (22%) non-MDR; 59 patients had ≥ 1 ACB infection (median of 2 [IQR 2-4] infections). Skin and soft-tissue infections (48%) were most common, followed by pneumonia (23%), bloodstream infections (15%), and osteomyelitis (11%) (Table 2). Compared to non-MDR ACB patients, patients with MDR ACB had greater transfusions requirements within 1^st^ 24 hours post-injury (median 25 vs 15 units p=0.01, Table 1). There was a non-significant trend toward more abdominal/pelvic/groin injuries among MDR ACB patients (60% vs 42% with non-MDR p=0.051). Hospital duration was a median 52.5 days and crude mortality was 3% with no significant difference between the groups.

**Conclusion:**

Battlefield casualties with ACB infections were characterized by high injury severity, frequently caused by blast trauma with MDR ACB more often in those with larger volume blood transfusions. Wound infections and pneumonia were most common. Overall, ACB infection patients were critically ill with large resource utilization regardless of MDR status. Further analysis to compare outcomes with non-ACB infection outcomes is warranted.

**Disclosures:**

**David Tribble, MD, DrPH**, AstraZeneca: The IDCRP and HJF were funded to conduct an unrelated phase III COVID-19 monoclonal antibody immunoprophylaxis trial as part of US Govt COVID Response

